# Economic incentives and diagnostic coding in a public health care system

**DOI:** 10.1007/s10754-016-9201-9

**Published:** 2016-10-14

**Authors:** Kjartan Sarheim Anthun, Johan Håkon Bjørngaard, Jon Magnussen

**Affiliations:** 1 Department of Public Health and General Practice, NTNU, Norwegian University of Science and Technology, 7491 Trondheim, Norway; 2 Department of Health Research, SINTEF Technology and Society, Trondheim, Norway; 3 Forensic Department and Research Centre Brøset, St. Olav’s University Hospital Trondheim, Trondheim, Norway

**Keywords:** Case-mix, DRG, DRG creep, Funding, Hospitals, Financing, I12, I13, I18, G38, D22, I10

## Abstract

We analysed the association between economic incentives and diagnostic coding practice in the Norwegian public health care system. Data included 3,180,578 hospital discharges in Norway covering the period 1999–2008. For reimbursement purposes, all discharges are grouped in diagnosis-related groups (DRGs). We examined pairs of DRGs where the addition of one or more specific diagnoses places the patient in a complicated rather than an uncomplicated group, yielding higher reimbursement. The economic incentive was measured as the potential gain in income by coding a patient as complicated, and we analysed the association between this gain and the share of complicated discharges within the DRG pairs. Using multilevel linear regression modelling, we estimated both differences between hospitals for each DRG pair and changes within hospitals for each DRG pair over time. Over the whole period, a one-DRG-point difference in price was associated with an increased share of complicated discharges of 14.2 (95 % confidence interval [CI] 11.2–17.2) percentage points. However, a one-DRG-point change in prices between years was only associated with a 0.4 (95 % CI $-1.1$ to 1.8) percentage point change of discharges into the most complicated diagnostic category. Although there was a strong increase in complicated discharges over time, this was not as closely related to price changes as expected.

## Introduction

A number of countries have introduced activity-based payment systems for hospital care by linking all or part of the hospital budget to the number of discharged patients while at the same time adjusting for treatment intensity or patient complexity (case mix). The diagnosis-related group (DRG) is one of the most common systems used to account for case mix. DRGs are widely used for both monitoring and payment purposes. The size of the reimbursement differs between patients, reflecting differences in complexity and thus treatment costs. Patients are categorized in different groups based on diagnosis and procedural codes routinely registered in medical records. For some groups, the DRG system makes the distinction between a “complicated” and an “uncomplicated” patient. While the main diagnosis will be the same, complicated patients will have one or more additional “complicating” secondary diagnoses. Within the resulting pair of DRGs, the complicated group will thus have higher predicted costs and a higher reimbursement. Because personnel in hospitals register information about diagnosis, there is the possibility that a patient is consciously coded to a “complicated” DRG. This is often referred to as “upcoding” or “DRG creep”, first defined as “a deliberate and systematic shift in a hospital’s reported case mix in order to improve reimbursement” (Simborg 1981). It has also been argued that the introduction of activity-based payment systems will increase the importance of accuracy and completeness in coding (Fisher et al. 1992; O’Reilly et al. 2012). The latter view is shared by the Norwegian government body responsible for the Norwegian DRG system, which defines DRG creep as “patients being coded as more complete, resulting in an increase in case mix index” (translated by the authors from Helsedirektoratet (2011)). Indeed, evidence from the US Medicare system indicated that the introduction of a prospective payment system in 1983 was followed by an increase in the average case mix (Carter and Ginsburg 1985; Ellis and McGuire 1986; Carter et al. 1990; Stern and Epstein 1985; Rosenberg 2001).

In the past decade, there has been a renewed interest in issues related to DRG creep and upcoding. Examining a policy reform in the financing of US Medicare discharges, (Dafny 2005) found a positive association between price differences between complicated and uncomplicated DRGs and the share of discharges in complicated groups. More recently, Barros and Braun (2016) found a positive association between price incentives and upcoding in Portugal.

Responses to price incentives vary between different types of hospitals. In Sweden, the increase in the number of secondary diagnoses registered was larger in hospitals with prospective payment systems than hospitals without prospective payment systems (Serdén et al. 2003). Two studies in the USA found that for-profit hospitals were more likely than non-profit or government-owned hospitals to upcode (Dafny and Dranove 2009; Silverman and Skinner 2004), and also that hospitals in “economic distress” were more likely to upcode (Silverman and Skinner 2004). However, no difference in upcoding between public and private hospitals was found in Italy (Berta et al. 2010).

In a cross-country comparative study, Steinbusch et al. suggest that health systems combining for-profit hospitals with the use of secondary diagnosis criteria for classification, such as in the USA, were more susceptible to upcoding (Steinbusch et al. 2007). In a systematic review, Palmer et al. argued that the effects seen in other countries are similar to those observed in the US system (Palmer et al. 2014). In a theoretical work, Kuhn and Siciliani suggested that the level of auditing of the financing system will influence the perceived risk related to upcoding, and this can also explain differences in levels of upcoding across health systems (Kuhn and Siciliani 2008).

The purpose of this paper is to add to the relatively small literature on upcoding in systems dominated by public hospitals by providing an analysis of coding behaviour in Norway over a period of 10 years. The Norwegian health care system is tax funded, with universal access to services that are largely free at the point of use. Hospitals are predominantly publicly owned and financed through a combination of global budgets and activity-based funding. Activity-based financing was introduced in 1997 utilizing a Nordic version of the DRG system. In the period covered by this study (1999–2008), the share of activity-based funding fluctuated between 40 and 60 %.^1^ The period also encompasses a major ownership reform in 2002, where hospital ownership was transferred from 19 county councils to the state (Magnussen et al. 2007).

Analysing coding behaviour in the Norwegian health care sector allowed us to address three questions. First, in a public health care system, the additional income generated from upcoding remains in the hospital. Thus, it will be used to increase the level of activity beyond what was planned, to increase slack (inefficiencies), or it will be saved to finance future investments. It remains uncertain to what extent actors in this public setting will seek to increase income by upcoding. Second, the substantial changes in the degree of activity-based funding during the period studied allowed us to analyse to what extent public hospitals adjust their coding behaviour *in response to changes* in financial incentives. Third, using observations over a period of 10 years allowed us to study any underlying trends in coding behaviour, and isolate this from the effects of changes in financial incentives. In all three questions, our main interest was the potential relationship between economic incentives and coding behaviour on an aggregate national level. Although there are numerous micro-level examples of upcoding (Lægreid and Neby 2012; Neby et al. 2015), it is unclear whether these are exceptions to the rule, or whether they represent a general behavioural response to economic incentives.

## Materials and methods

### Data material

Data from all Norwegian somatic hospital discharges for the period 1999–2008 were used. The Norwegian Patient Registry provided the data.^2^ Each hospital discharge was grouped in a DRG, and 250 of the total of 913 groups were linked in complicated/uncomplicated pairs (in 2008). Only patients in acute care hospitals grouped within these 125 DRG pairs were included. We excluded DRG pairs not used in all years, DRG pairs with fewer than 1000 annual cases, and five additional DRG pairs that were viewed as problematic.^3^ After exclusion criteria were applied, 3,180,578 in-patient discharges remained. They were grouped into 76 different DRG pairs, of which 53 pairs were medical DRGs and 23 pairs were surgical DRGs. These pairs amount to about 29 % of the total volume of discharges. See Table 1 for a list of included DRG pairs. Our study included 26 hospitals (including three large publicly funded non-profit private hospitals). Not all hospitals treated patients in all included DRGs.

**Table 1:** List of DRGs included in study

DRG code	DRG text	M/S	% compl.	# disch. (1000)	Case-mix adjusted # disch. (1000)	$\overline{p_i}$	Mean absolute $\Delta p_{it}$
10	Nervous system neoplasm	M	43.1	33.7	42.8	0.379	0.068
18	Cranial and periferal nerve disorders	M	27.5	22.3	17.5	0.230	0.042
24	Seizure and headache age >17	M	25.9	77.9	42.0	0.223	0.075
31	Concussion, age >17	M	16.5	44.4	12.9	0.051	0.022
34	Other disorders of nervous system	M	23.8	78.4	62.3	0.257	0.069
46	Other disorders of the eye, age >17	M	25.3	21.1	10.1	0.256	0.051
68	Otitis media and uri, age >17	M	25.6	24.7	11.7	0.138	0.024
70	Otitis media and uri, age 0–17	M	14.7	34.3	12.3	0.143	0.057
79	Respiratory infections and inflammations, age >17	M	67.7	29.8	61.1	0.390	0.049
89	Simple pneumonia and pleurisy, age >17	M	71.4	186.5	264.5	0.310	0.037
91	Simple pneumonia and pleurisy, age 0–17	M	23.2	18.1	14.4	0.343	0.069
96	Bronchitis and asthma, age >17	M	37.8	25.7	20.3	0.184	0.030
98	Bronchitis and asthma, age 0–17	M	10.1	48.7	28.8	0.204	0.041
99	Respiratory signs and symptoms	M	25.9	26.0	10.9	0.172	0.042
101	Other respiratory system diagnoses	M	40.1	13.3	9.6	0.220	0.029
110	Major cardiovascular procedures	S	55.7	18.2	82.1	0.467	0.179
124	Diagnostic percutan cardiac procedure w circulatory complex dx	M	31.8	33.7	19.0	0.187	0.044
130	Peripheral vascular disorders	M	46.1	58.0	49.1	0.194	0.036
132	Atherosclerosis	M	57.9	43.8	26.9	0.144	0.013
135	Cardiac congenital and valvular disorders age >17	M	73.0	19.1	16.4	0.208	0.066
138	Cardiac arrythmia and conduction disorders	M	35.5	123.9	56.7	0.170	0.033
141	Syncope and collapse	M	35.5	49.8	21.5	0.078	0.013
144	Other circulatory system diagnoses	M	53.7	23.1	21.4	0.243	0.056
146	Rectal resection	S	54.8	11.9	43.8	0.552	0.149
148	Major small and large bowel procedures	S	59.8	46.6	173.8	0.769	0.158
157	Minor intestinal procedure	S	17.0	30.8	20.0	0.361	0.050
159	Hernia procedures except inguinal and femoral, age >17	S	25.4	12.5	11.3	0.361	0.086
161	Inguinal and femoral hernia procedures, age >17	S	26.0	22.7	14.9	0.154	0.067
170	Other digestive system o. r. procedures	S	40.8	14.2	30.0	0.711	0.170
172	Digestive malignancy	M	68.4	78.6	88.0	0.204	0.047
174	G. i. hemorrhage	M	57.5	51.6	43.1	0.202	0.029
177	Uncomplicated peptic ulcer	M	44.0	10.3	7.6	0.212	0.076
180	G. i. obstruction	M	41.4	15.3	8.5	0.182	0.037
182	Esophagitis, gastroent and misc digest disorders, age >17	M	30.4	249.1	116.0	0.137	0.020
184	Esophagitis, gastroent and misc digest disorders, age 0–17	M	15.8	71.0	26.2	0.103	0.028
188	Other digestive system diagnoses, age >17	M	36.4	41.0	22.7	0.237	0.024
205	Disorders of liver except malig, cirr, alc hepa	M	41.3	17.9	17.8	0.367	0.110
207	Disorders of biliary tract	M	35.1	49.1	36.5	0.243	0.043
210	Hip and femur procedures except major joint, age >17	S	54.9	92.5	189.5	0.302	0.092
218	Lower extrem and humer proc except hip, foot, femur age >17, with cc	S	19.5	55.9	77.4	0.668	0.119
221	Knee procedures	S	13.6	35.8	38.6	0.696	0.172
223	Major shoulder/elbow proc, or other upper extremity proc	S	13.8	56.2	49.8	0.283	0.048
226	Soft tissue procedures	S	12.4	29.5	21.9	0.421	0.042
228	Major thumb or joint proc, or oth hand or wrist proc	S	22.8	29.1	18.0	0.192	0.087
244	Bone diseases and specific arthropathies	M	37.2	22.1	15.8	0.179	0.028
250	Fracture, sprain, strain or dislocation of forearm, hand or foot, age >17	M	24.2	14.9	5.1	0.214	0.040
253	Fracture, sprain, strain or dislocation of upper arm or lower leg excluding foot, age>17	M	25.5	41.9	22.4	0.234	0.035
257	Total mastectomy for malignancy	S	33.2	15.1	18.2	0.110	0.026
259	Subtotal mastectomy for malignancy	S	22.1	16.2	13.9	0.116	0.011
269	Other skin and subcut tiss proc	S	34.3	21.6	21.2	0.610	0.055
272	Major skin disorders	M	54.5	17.7	24.0	0.307	0.127
277	Cellulitis age >17	M	39.0	45.6	41.7	0.217	0.016
280	Trauma to the skin and subcut tiss age >17	M	34.4	39.9	16.3	0.153	0.021
283	Minor skin disorders	M	25.7	24.2	17.8	0.246	0.074
296	Nutritional and misc metabolic disorders, age >17	M	53.5	27.8	21.9	0.193	0.027
300	Endocrine disorders	M	38.3	20.7	15.6	0.241	0.035
308	Minor bladder procedures	S	26.9	18.9	24.3	0.395	0.278
310	Transurethral procedures	S	37.1	36.3	29.8	0.170	0.040
318	Kidney and urinary tract neoplasms	M	69.6	25.5	31.6	0.365	0.073
320	Kidney and urinary tract infections age >17	M	53.4	71.5	65.8	0.182	0.023
323	Urinary stones,&/or esw lithotripsy	M	29.2	44.9	23.2	0.125	0.031
325	Kidney and urinary tract signs and symptoms age >17	M	45.8	19.9	9.4	0.108	0.020
331	Other kidney and urinary tract diagnoses age >17	M	47.0	18.2	13.3	0.281	0.070
336	Transurethral prostatectomy	S	40.9	37.4	40.1	0.137	0.020
346	Malignancy, male reprocuctive system	M	72.9	43.5	42.5	0.200	0.056
358	Uterine and adnexa proc for ovarian or adnexal non-malignancy	S	14.4	66.6	90.6	0.429	0.080
366	Malignancy, female reproductive system	M	60.7	47.1	54.2	0.367	0.059
370	Cesarean section	S	31.0	87.5	126.2	0.295	0.069
383	Other antepartum diagnoses w medical complications	M	56.8	56.4	27.0	0.112	0.014
398	Reticuloendothelial and immunity disorders	M	40.5	14.7	14.3	0.320	0.066
403	Lymphoma and non-acute leukemia	M	54.2	72.2	96.6	0.529	0.054
442	Other o. r. procedures for injuries	S	52.2	10.4	28.7	1.192	0.294
444	Traumatic injury, age >17	M	34.9	10.8	5.5	0.241	0.033
449	Poisoning and toxic effects of drugs, age >17	M	29.2	55.2	18.7	0.155	0.043
463	Signs and symptoms	M	36.6	16.0	11.6	0.179	0.042
493	Laparoscopic cholecystectomy w/o c. d. e.	S	25.3	43.8	80.0	0.262	0.043

DRG code and DRG text is for complicated group in the pair

M/S: M $=$ Medical DRG pair, S $=$ Surgical DRG pair

% compl: Percentage of complicated discharges in pair. Defined as number of complicated discharges divided by total number of discharges

# disch: Number of inpatient discharges in DRG pair, 1000

Case-mix adjusted # disch: Case-mix adjusted number of inpatient discharges in DRG pair, 1000 (adjusted by the weights used for reimbursements)

$\overline{p_i}:$ Mean difference in prices of complicated and uncomplicated group in pair

Mean absolute $\Delta p_{it}$: Mean absolute deviation from$\overline{p_i}$. Since the mean deviation from the mean in a group always is zero, we have here showed the mean absolute deviation in this table

### Dependent variable

The dependent variable ($$c_{tih} )$$ was the percentage of complicated discharges in a DRG pair. This was defined as the number of complicated cases divided by the total number of cases in the DRG pair, calculated for year *t*, DRG pair *i* and hospital *h*.

### Potential gain in income from upcoding: the incentive

We measured the potential gain in income from upcoding as the difference in reimbursement (DRG prices) between complicated and uncomplicated groups in each DRG pair similarly to the *spread* in weights as defined by Dafny (2005) and Barros et.al. (Barros and Braun 2016). This spread did not differ across hospitals, as there were no hospital-specific prices. We calculated the difference between prices of complicated and uncomplicated groups within a DRG pair across the years, multiplied by the share of activity-based funding for each specific year. However, we depart from Dafny’s approach by calculating the mean across years for each DRG pair and denote this as $\overline{p_i}$ (Eq. 1). To enable comparison across years, we measured prices normalized in DRG points, not as the monetary value of a DRG point. One DRG point, roughly equalling the treatment cost of the “average patient”, was valued at 33,647 NOK ($\sim$3629 EUR) in 2008. This should be interpreted as the incentive in a DRG pair because it increases income without increasing cost, should any upcoding take place.

1 $$\begin{aligned} \overline{p_i } =\frac{1}{10}*\mathop \sum \nolimits _{t=1999}^{2008} \Big ({\textit{COMPLICATED}_{it} -\textit{UNCOMPLICATED}_{it} }\Big )*\textit{ABFSHARE}_t \end{aligned}$$

In Eq. 1, *COMPLICATED* $_{it}$ is the DRG weight (relative price) of the complicated group in DRG pair *i* in year *t*, *UNCOMPLICATED* $_{it}$ is the DRG weight of the uncomplicated group in DRG pair *i* in year *t* and *ABFSHARE* $_{t}$ is the share of the total budget allocated through activity-based financing (from 0 to 1) in year *t*.

However, the price of each DRG may change from year to year. Such changes are caused by (1) changes in relative reimbursement rates (prices are adjusted annually) for specific DRGs (i.e., *COMPLICATED* $$_{it}$$ and *UNCOMPLICATED* $$_{it})$$, and (2) variations in the share of activity-based funding between years (*ABFSHARE* $$_{t})$$. Either of these causes will yield changes in the potential gain in income. In this study, we are not only interested in the level of the incentive, ($$\overline{p_i})$$, but also in changes calculated as the annual changes from the average for each DRG pair (Eq. 2).

2 $$\begin{aligned} \Delta p_{it} =\Big ({\Big ({\textit{COMPLICATED}_{it} -\textit{UNCOMPLICATED}_{it}} \Big )*\textit{ABFSHARE}_t}\Big )-\overline{p_i} \end{aligned}$$

By separating $\overline{p_i}$ and $\Delta p_{it}$, we separate the effect of the *level* of the incentive from *changes* in the incentive on coding behaviour. The level of the incentive is thus the difference *between* DRG pairs ($\overline{p_i})$, while the changes are differences over time *within* a specific DRG pair ($\Delta p_{it} )$. The spread used by Dafny (2005) and Barros et.al. (Barros and Braun 2016) is the sum of these between and within effects.

### Statistical analysis

The clustered and hierarchical nature of the data led us towards a mixed-model approach. The multivariable analyses were performed using a three-level linear regression model, where hospital discharges were aggregated to 19,250 observations, comprising 10 yearly observations (level 1) of each DRG pair (level 2) within each of the 26 hospitals (level 3). Equation 3 describes our main analytical model.

3 $$\begin{aligned} c_{tih} =a+a_i +a_h +b_1 \overline{p_i}+b_2 \varDelta p_{it}+b_3 T_t +b_4 D+b_5 T_t D+b_x x_{tih} +\varepsilon _{tih} \end{aligned}$$

Our dependent variable, $$c_{tih}$$, is the share of complicated cases in year *t* in DRG pair *i* in hospital *h*. The effects of the level of the upcoding incentive were defined by $$\overline{p_i}$$ (Eq. 1), and the change in incentive defined by $$\Delta p_{it} $$ (Eq. 2). To capture any general development in coding practice over time, we included time trend ($$T_{t})$$, which measures years since 1999. This time trend might, however, capture both general improvements in quality of coding, as well as any fraudulent upcoding not captured by the effects of $$\overline{p_i}$$ and $$\Delta p_{it} $$. We also controlled (by way of a dummy (*D*) for the years 2002–2008) for the possible effect of the ownership reform in 2002. A statistical interaction of these was included ($$T_{t}D)$$.

The *a*-terms are constants and intercepts at the different levels while $\varepsilon _{tih}$ is the residual. Other covariates are denoted $x_{tih}$ in the equation. These included average age and sex in each DRG pair. Elderly patients are more likely to be frailer, and therefore have an increased probability of being grouped in complicated groups.^4^ For the same reason, we also adjusted for emergency status and length of stay. Emergency admissions are more likely to be complicated than elective procedures (Melnick et al. 1989; Keller et al. 1987). Length of stay may be a proxy for case mix as the longer the patient remains in the hospital, the more complex the illness is likely to be or the frailer the patient. To better control for co-morbidity and case mix, we constructed a Charlson index for each analytical observation. The index is a measure of co-morbidity that is based upon secondary diagnoses (Charlson et al. 1987), as also was our dependent variable. For the calculation of the Charlson index, we excluded those diagnoses that caused a complicated DRG grouping (within each DRG pair), and thus the index does not have an upcoding bias other than what comes from the complicated discharges actually being more complicated.

While ownership of hospitals after 2002 was transferred to the state, there was an administrative decentralization to four regional health authorities. The regional health authorities face different challenges, as there are substantial differences in distance to hospital, different degrees of deficits/surpluses and also size of population. We also included dummy variables for these to account for possible regional variances in coding behaviour induced by diverse organizational incentives or structures. The annual number of in-patient treatments at each hospital (measured as case mix-adjusted DRG points) was included as a proxy for hospital size. This measure will be invariant at the DRG pair level. Finally, we performed a stratified analysis of medical and surgical DRGs, because surgical DRGs could arguably have less room for differences in coding behaviour than medical DRGs. Precision was estimated with 95 % confidence intervals (CI).

Even though the dependent variable is a proportion, we assumed normality in the residuals. Robustness tests were performed with a simpler two-level model, using the actual monetary value as main independent variables instead of the rather abstract DRG points.

## Results

### Descriptive statistics

**Fig. 1:**
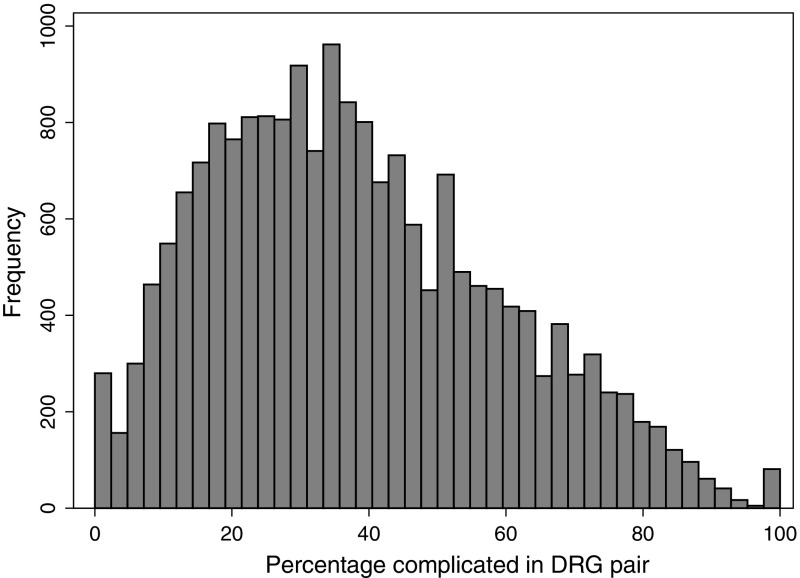
Distribution of percentage complicated in DRG pair, histogram

**Fig. 2:**
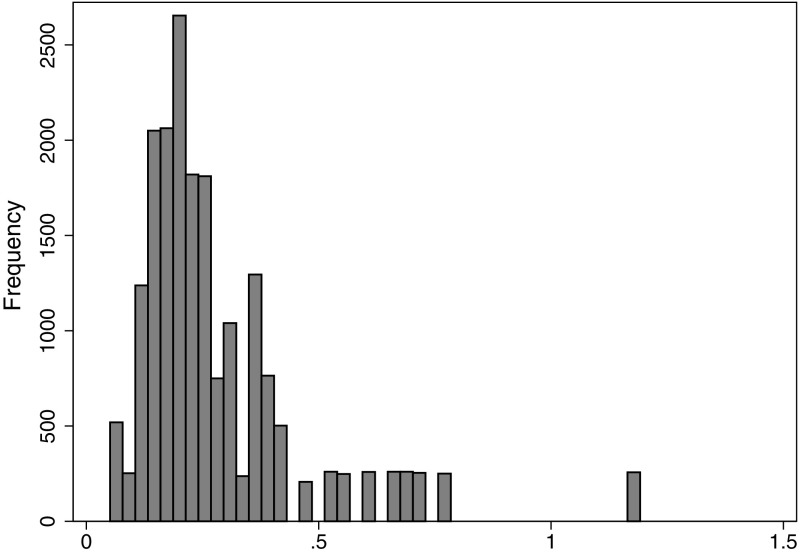
Distribution of potential gain in income $\overline{p_i}$, histogram

**Fig. 3:**
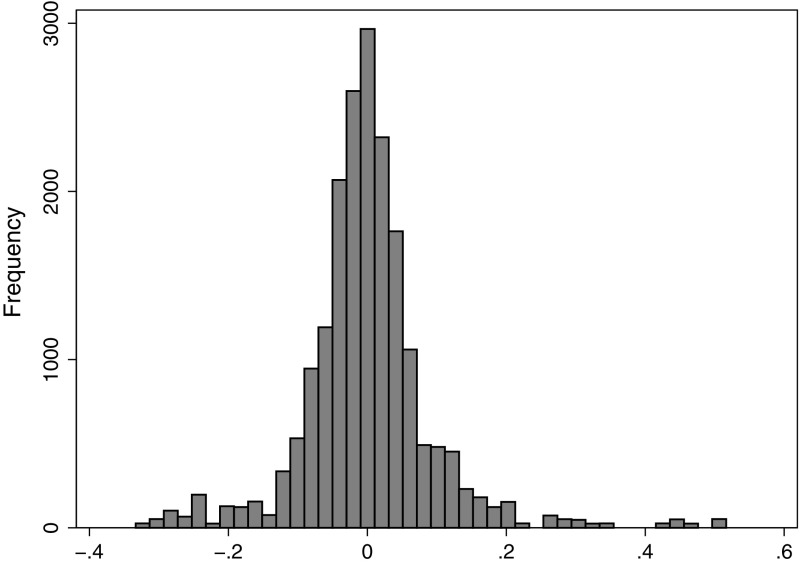
Distribution of changes in potential gain in income $\Delta p_{it}$, histogram

Table 2 presents descriptive statistics. Across the observations (year, DRG pair, hospital), the mean share of complicated discharges was 38 %, ranging from 0 to 100 (see Fig. 1 for distribution). The mean $\overline{p_i}$ was 0.28 DRG points and ranged from 0.05 to 1.19 (see Fig. 2 for distribution). The mean change ($\Delta p_{it}$) was zero because this was defined as yearly deviations from $\overline{p_i}$. Table 1 lists $\overline{p_i}$ and the mean absolute $\Delta p_{it}$ for each DRG pair, and Fig. 3 shows the distribution of $\Delta p_{it}$.

**Table 2:** Descriptive statistics for variables in analysis

Variable	Mean	Median	Std. Dev.	Min	Max
Age	55.57	58.16	1.59	1.00	98.00
Percentage female	51.19	49.70	21.09	0.00	100.00
Percentage emergency	70.75	81.24	29.16	0.00	100.00
Length of stay	4.87	4.10	3.10	0.00	46.00
Number of inpatient treatments at hospital*	11,496	8959	8383	1812	43,540
Percentage medical DRGs	70.20	100.00	45.73	0.00	100.00
Charlson co-morbidity index	0.26	0.18	0.33	0.00	8.00
Potential gain in income $\overline{p_i}$	0.28	0.23	0.18	0.05	1.19
Changes in potential gain in income $\Delta p_{it}$	0.00	$-$0.00	0.09	$-$0.33	0.52
Percentage complicated discharges ($$c_{tih})$$	38.01	35.30	20.94	0.00	100.00

*N* = 19,250

* Case-mix adjusted, DRG-pair invariant

Data analysis was performed at an aggregate level, i.e., the mean age of 55.6 was the mean across all observations (year, DRG pair, hospital) and not the mean for all distinct patients. On average, the share of females was 51.2 %, but this varied from 0 to 100 as some DRG pairs were gender specific. The mean length of stay was 4.87, but varied across DRG pairs with a maximum of 46. Some DRG pairs had a zero length of stay and were thus likely to be patients admitted as in-patients but discharged on the same day. There was a downward trend in length of stay over the period. To control for hospital size, we also calculated the (case mix-adjusted) number of in-patient discharges at each hospital. This was measured annually at the hospital level, and as opposed to the other independent variables, this was DRG pair invariant. Hospital size varied substantially with the mean of 11,496 discharges while the largest hospital had 43,540 discharges. Mean hospital size also increased over the period covered by this study, both through reforms and reorganizations/mergers as well as increased budgets. All control variables were centred on their mean in the multivariable analysis.

### Multivariable analysis

Table 3 shows the correlations between the variables of interest. The share of complicated discharges ($$c_{tih})$$ was highly correlated with the case mix-related variables: age (Pearson’s *r* correlation coefficient 0.512), length of stay (0.461) and comorbidity (0.510). The share of complicated discharges was also positively correlated with the temporal variables, emergency admissions and medical DRG pairs. At this aggregate level, there was a small yet statistically significant association with $$\overline{p_i}$$ (0.091), but not with $$\Delta p_{it}$$.

**Table 3:** Correlation matrix of share complicated discharges and all independent variables

	Percentage complicated discharges $c_{tih}$	$\overline{p_i}$	$\Delta p_{it}$	Time trend ($\hbox {T}_{t}$)	Reform (shift 2002–2008)	Interaction time trend and reform	Age	Share female patients	Share emergency admissions	Length of stay	Hospital size	Medical DRG pairs (dummy)
$\overline{p_i}$	0.091*											
$\Delta p_{it}$	0.005	0.000										
Time trend (T$$_{t})$$	0.246*	$$-$$0.000	$$-$$0.058*									
Reform (shift 2002–2008)	0.258*	$-$0.000	0.011	0.798*								
Interaction time trend and reform	0.249*	$-$0.000	$-$0.0482*	0.984*	0.854*							
Age	0.512*	$-$0.003	$-$0.001	0.022*	0.021*	0.022*						
Share female patients	$-$0.064*	0.007	0.003	$-$0.003	0.002	$-$0.003	$-$0.099*					
Share emergency admissions	0.163*	$-$0.215*	$-$0.005	0.048*	0.030*	0.047*	$-$0.116*	$-$0.059				
Length of stay	0.461*	0.514*	0.032*	$-$0.116*	$-$0.101*	$-$0.114*	0.347*	0.051*	-0.146*			
Hospital size	0.015*	0.001	$-$0.001	0.070*	0.072*	0.070*	$-$0.099*	$-$0.017*	$-$0.118*	0.010		
Medical DRG pairs (dummy)	0.197*	$-$0.497*	$-$0.000	0.006	0.004	0.006	$-$0.035*	$-$0.091*	0.650*	$-$0.224*	$-$0.013	
Mean Charlson index	0.510*	0.0737*	0.006	0.154*	0.140*	0.153*	0.355*	$-$0.053*	0.018*	0.354*	0.026*	0.100*

$$*\,p<0.05$$

In the multilevel regressions, there was a positive association between $\overline{p_i}$ and the share of complicated discharges (Table 4). Over the whole period, a one-DRG-point difference in $\overline{p_i}$ was associated with an increased share of complicated discharges of 14.2 percentage points (95 % CI 11.2–17.2). However, a one-DRG-point change in $\Delta p_{it}$ between years was only associated with an increase of the most complicated group of 0.4 percentage points (95 % CI $-1.1$ to 1.8).

**Table 4:** Multilevel linear regression of the percentage of complicated discharges, coefficients with 95 % CI in parenthesis

	Complete model	Only surgical DRG pairs	Only medical DRG pairs
Potential gain in income $\overline{p_i}$	14.23*** (11.23 to 17.24)	17.08*** (14.21 to 19.95)	13.19*** (6.09 to 20.29)
Changes in potential gain in income $\Delta p_{it}$	0.35 ($-$1.10 to 1.79)	$-$2.45*** ($-$4.27 to $-$0.62)	5.08*** (2.54 to 7.63)
Time trend (years since 1999)	2.85*** (2.58 to 3.12)	3.04*** (2.52 to 3.57)	2.85*** (2.54 to 3.17)
Reform (dummy for years 2002–2008)	10.23*** (9.64 to 10.81)	9.66*** (8.55 to 10.77)	10.60*** (9.92 to 11.27)
Interaction time trend and reform	$-$2.41*** ($-$2.68 to $-$2.13)	$-$2.49*** ($-$3.02 to $-$1.96)	$-$2.42*** ($-$2.74 to $-$2.10)
Ten percentage points increase in women	$-$0.22*** ($-$0.35 to $-$0.09)	$-$0.41*** ($-$0.60 to $-$0.21)	$-$0.20** ($-$0.36 to $-$0.04)
Ten percentage points increase in emergency admissions	0.96*** (0.83 to 1.09)	0.62*** (0.42 to 0.81)	1.15*** (0.96 to 1.33)
Length of stay	1.25*** (1.16 to 1.35)	1.29*** (1.14 to 1.43)	1.25*** (1.12 to 1.38)
Hospital size (case-mix adjusted number of inpatient treatments/1000)	0.55*** (0.42 to 0.68)	0.38*** (0.22 to 0.53)	0.42*** (0.29 to 0.55)
Medical DRG pairs compared with surgical	8.09*** (6.78 to 9.40)		
Charlson index	12.54*** (11.74 to 13.34)	10.44*** (8.91 to 11.97)	13.59*** (12.66 to 14.53)
*N*	19,250	5,736	13,514

$$^{***}\,p<0.01, ^{**}\,p<0.05, ^{*}\,p<0.1$$. Controlled for regional health authorities (with dummies) and five age splines. Random effects of time trend, otherwise fixed effects

The temporal variables had large estimated values. There was a large annual increase in the share of complicated discharges of 2.9 percentage points (95 % CI 2.6–3.1) in the period leading up to the reform (1999–2001). After the reform in 2002, there was a shift in the share of complicated discharges of 10.2 percentage points (95 % CI 9.6–10.8). By calculating the combined estimates of $T_{t}, \, D$ and $T_{t}D$, we find an annual increase of only 0.4 percentage points in the period after 2002.

The case-mix adjustors had a large impact on the share of complicated discharges. A one-unit increase in the Charlson index, which can be interpreted as one more co-morbidity, was associated with an increase of 12.5 percentage points in the share of complicated discharges. For an increase in mean length of stay of one day, the share of complicated discharges increased 1.3 percentage points (95 % CI 1.2–1.4). We found only a small negative association between share of females and percentage of complicated discharges. There were no substantial differences between the different regional health authorities. Hospital size had a small positive effect, indicating that larger hospitals have a higher share of complicated discharges.

The share of complicated discharges was 8.1 percentage points (95 % CI 6.8–9.4) higher in medical DRG pairs than in surgical DRG pairs. We performed a stratified analysis of medical and surgical DRG pairs. For medical pairs, a one-DRG-point change in $\Delta p_{it}$ was associated with an increase in share of complicated discharges of 5.1 percentage points (95 % CI 2.5–7.6) (Table 4); for the surgical DRG pairs, there was a negative effect from $\Delta p_{it}$ of $-2.5$ (95 % CI $-4.3$ to $-0.6$). Aside from the effect of $\Delta p_{it}$, there were no other large differences between the stratified and the non-stratified analyses.

Robustness tests were performed using simpler two-level models (either hospital level or DRG pair level), but the results did not differ much from the results presented in Table 4. We also ran the analysis using potential income gain measures calculated from the monetary refund that the hospitals received instead of DRG points. The refund was calculated using the yearly refund value of a DRG point while deflating the older years to real 2008 prices. The results did not differ much from the presented results. The test showed that for every 1000 NOK ($\sim$109   EUR) in increased potential income ($\overline{p_i})$, the share of complicated discharges increased by 0.31 percentage points. Nonetheless, changes in $\Delta p_{it}$ had no effect. Table 5 shows the different models tested for robustness.

**Table 5:** Multilevel linear regression of the percentage of complicated discharges; robustness test of other specifications: two level model combined DRG pairs and hospital, two level model DRG pairs, and monetary value of price incentive and changes in price incentive, $N=19,250$

	Two level model hospital	Two level model DRG pairs	Monetary value
Potential gain in income $\overline{p_i}$	10.67*** (9.27 to 12.06)	15.21** (2.99 to 27.43)	0.31*** (0.24 to 0.38)
Changes in potential gain in income $\Delta p_{it}$	$-$0.09 ($-$1.98 to 1.81)	0.73 ($-$1.23 to 2.68)	0.02 ($-$0.02 to 0.05)
Time trend (years since 1999)	3.11*** (2.62 to 3.59)	2.88*** (2.51 to 3.26)	2.98*** (2.71 to 3.26)
Reform (dummy for years 2002–2008)	10.64*** (9.78 to 11.50)	10.56*** (9.87 to 11.26)	10.48*** (9.89 to 11.06)
Interaction time trend and reform	$-$2.51*** ($-$2.92 to $-$2.10)	$-$2.48*** ($-$2.81 to $-$2.14)	$-$2.45*** ($-$2.72 to $-$2.17)
Ten percentage points increase in women	0.28*** (0.19 to 0.37)	$-$0.36*** ($-$0.51 to $-$0.20)	$-$0.22*** ($-$0.35 to $-$0.09)
Ten percentage points increase in emergency admissions	0.71*** (0.62 to 0.79)	0.85*** (0.75 to 0.96)	0.96*** (0.83 to 1.10)
Length of stay	1.99*** (1.91 to 2.08)	1.16*** (1.07 to 1.25)	1.26*** (1.17 to 1.36)
Hospital size (case-mix adjusted number of inpatient treatments/1000)	0.29*** (0.14 to 0.44)	0.09*** (0.07 to 0.11)	0.55*** (0.43 to 0.68)
Medical DRG pairs compared with surgical	10.44*** (9.83 to 11.05)	7.22*** (2.32 to 12.13)	7.91*** (6.61 to 9.20)
Charlson index	9.77*** (9.13 to 10.41)	16.27*** (15.42 to 17.11)	12.57*** (11.77 to 13.37)

$$^{***}\,p<0.01, ^{**}\,p<0.05, ^{*}\,p<0.1$$. Controlled for regional health authorities (with dummies) and five age splines. Random effects of time trend, otherwise fixed effects

## Discussion

Our goal was to examine the association between the potential gain in income from upcoding and the coding behaviour of hospitals. Across DRG pairs, we found a positive association between the gain in income from upcoding and the share of discharges classified as complicated. Thus, DRG pairs in which there was a higher gain in income from upcoding also had a higher share of complicated discharges. However, although we controlled for co-morbidity, age and length of stay, we cannot exclude the possibility that this partly reflects differences in the case mix. Nevertheless, it is not clear why the difference in treatment costs between complicated and uncomplicated discharges should be higher in DRG pairs with a higher share of complicated discharges and therefore our results indicate that coding behaviour is related to the size of the incentive.

We found that a difference in price between a complicated and uncomplicated group of one DRG point was related to a difference of 14 percentage points in the share of complicated discharges within a DRG pair. Although this may seem like a large effect, the average potential gain from upcoding was only 0.28 DRG points (see Table 2).

We found no association between changes in $\Delta p_{it}$ over time and the share of complicated discharges within a DRG pair. Thus, in a period with frequent changes in the share of activity-based funding, hospitals did not seem to respond by changing their coding behaviour. However, when stratifying the analysis by medical and surgical DRGs, we found a small, positive association for medical DRGs. Because surgical patients are generally more homogeneous (within a DRG) than medical patients, there may have been less opportunity for tactical coding of these patients. Although the size of the estimated association was small, this result indicated that there might be subgroups of patients where the relationship between financial incentives and tactical coding is stronger. This corresponds to earlier results on how Norwegian hospitals respond to price changes (Januleviciute et al. 2016). Melberg et al. have recently shown higher growth in DRG groups with a price increase than in groups with a reduction in reimbursement rates (Melberg et al. 2016).

We found that the share of complicated discharges increased during the ten year period covered by the study. This may be due to changes in case mix resulting from demographic changes, changes in technology, changes in the quality and completeness of coding and finally changes in the financing system. Recalling the two different definitions of upcoding and DRG creep presented in the introduction, we cannot here distinguish between “deliberate upcoding” and “more complete coding”. The increasing trend could both indicate that the quality of coding has improved, and at the same time that the presence of explicit and implicit incentives is followed by a general increase in the recording of secondary diagnoses. Thus, while we cannot label all upcoding as being completely driven by financial incentives, we argue that such incentives were present and that their consequences are reflected on an aggregate level by the increasing time trend. The introduction of activity-based funding in 1997 was followed by an increased use of secondary diagnoses. Eventually the use of secondary diagnoses will reach a level (or equilibrium) where it might be difficult to justify an additional secondary diagnosis from a medical point of view. Thus, one might suspect that a large part of the potential for increase was exhausted in the period following the hospital reform, explaining the slowing growth in the share of complicated discharges.

This paper decomposed the price incentive into two components, $\overline{p_i}$ and $\Delta p_{it}$, to differentiate between the level and changes of the incentive for upcoding. This approach differs from earlier studies but demonstrates that, in Norway, the differences in prices are more important than changes within groups. Hospitals may appear to respond to prices, but the changes in price are probably too small to have a large-scale impact.

We believe that the major strength of this analysis is the fact that we are able to utilize a complete dataset covering all DRG pairs for all patients at all hospitals. Our analyses include a ten year period in which there have been large and repeated changes in the potential gain in income from upcoding. Thus, any aggregate effects of increased gain in income from upcoding should be detected in this study. By controlling for a time trend and separating within and between effects, we are more reassured that any remaining effects are more related to upcoding rather than to an increase in the quality of coding.

We have employed a system perspective by pooling all DRG pairs, hospitals and years in the same analysis. This could dilute important findings for specific DRG pairs. Silverman and Skinner (2004) found substantial evidence of upcoding for patients with pneumonia. Their results were robust to different model specifications, but sensitive to the included DRGs. Our stratification showed very different results for the medical and surgical DRG pairs. It is safe to assume that even larger differences will be found on examination of separate DRGs. However, our aim was to detect system-level effects and not effects of singular groups or hospitals. One might also question whether the observed changes in the price incentive were large enough to have an effect. While frequent and potentially substantial, the changes in incentives observed in this study were small compared with some of the larger exogenous shocks described by, for example, Dafny (2005). Therefore, it may have been unrealistic to expect significant results from the observed changes. A change of 20 percentage points in the share of activity-based funding is, however, not trivial and it is interesting that these changes only seem to have led to a marginal change in coding practice.

Upcoding can take place in all systems that incentivize documenting of diagnoses. We have limited our study to upcoding in DRG pairs in Norway. These groups amount to less than one-third of the total volume of treatment. Upcoding is possible for all groups, but the paired structure of complicated/uncomplicated lends itself easily to our research strategy of testing directly whether incentives are associated with upcoding. There are several ways “manipulations” can occur in a DRG system (Neby et al. 2015). In this paper, we have focused solely on upcoding and not touched upon other related strategies: gaming, dumping, skimping and skimming. Further studies should attempt to distinguish upcoding from other manipulations empirically. It is impossible using registry data to determine whether the upcoding has been deliberate. To assess the actual conscious decision to upcode, one must opt for a qualitative approach. This study has not ventured into the auditing of diagnosis and hospital records. Earlier evidence from Norway has indicated that diagnostic accuracy is not very high (Jørgenvåg 2005), and it would be interesting to consider whether the Norwegian auditing scheme could be considered optimal (Kuhn and Siciliani 2008).

## Footnotes

1 In 1999–2001, the share of income related to activity was 50 %, increasing to 55 % in 2002 and 60 % in 2003. The share fell to 40 % in 2004, and rose again to 60 % in 2005. The share returned to 40 % in the years 2006–2008.

2 The Norwegian Patient Register is a complete registry of all specialized hospital care. The interpretation and reporting of these data are the sole responsibility of the authors, and no endorsement by the Norwegian Patient Register is intended nor should be inferred.

3 These five excluded DRGs were 372/373 (Vaginal births), 76/77 (Other respiratory operating room procedures), 452A/453A (Complications of treatment with surgery), 454/455 (Other injury, poisoning & toxic effect) and 478/479 (Other vascular procedures). Among these DRG pairs, vaginal births was the largest of all complicated/uncomplicated pairs, and was excluded due to significant alterations in the specifications of the DRG pair during the period.

4 In the regressions, we control for age by restricted cubic splines, calculated with five knots (Harrell 2001). Five knots means that the age range is split in five groups. These splines provide a better control and fit of variables than a simple linear approach. However, the resulting coefficients are not readily interpretable as they are not marginal linear effects.
